# Up-regulated S100 calcium binding protein A8 in *Plasmodium*-infected patients correlates with CD4^+^CD25^+^Foxp3 regulatory T cell generation

**DOI:** 10.1186/s12936-015-0855-4

**Published:** 2015-10-05

**Authors:** Hyeong-Woo Lee, Tong-Soo Kim, Yoon-Joong Kang, Jung-Yeon Kim, Sangeun Lee, Won-Ja Lee, Youngjoo Sohn

**Affiliations:** Department of Parasitology and Tropical Medicine, Inha Research Institute for Medical Sciences, Inha University School of Medicine, Incheon, 400-712 Republic of Korea; Department of Biomedical Science, Jungwon University, Goesan, Chungbuk 367-805 Republic of Korea; Division of Malaria and Parasitic Disease, Korea National Institute of Health, Osong, 363-951 Republic of Korea; Department of Pathology, Immunology, and Laboratory Medicine, College of Medicine, University of Florida, Gainesville, FL 32610 USA; Division of Arbovirus, Korea National Institute of Health, Osong, 363-951 Republic of Korea; Department of Anatomy, College of Korean Medicine, Institute of Korean Medicine, Kyung Hee University, Seoul, 130-701 Republic of Korea

## Abstract

**Background:**

The pro-inflammatory S100 calcium binding protein A8 (S100A8) is elevated in the serum of patients with *Plasmodium falciparum* malaria, but its function in *Plasmodium vivax* malaria is not yet clear. This function was investigated in *P. vivax*-infected patients in this study.

**Methods:**

The level of S100A8 in the serum was measured with ELISA. Full amino acids of S100A8 were synthesized to verify the functions for maturation of immature dendritic cell (iDC) and evaluation of CD4^+^CD25^+^Foxp3^+^ regulatory T (Treg) generation by mature DC (mDC).

**Results:**

A higher amount of S100A8 was detected in vivax-infected patients (141.2 ± 61.849 ng/ml, n = 40) compared with normal control group (48.1 ± 27.384 ng/ml, n = 40). The level of S100A8 did not coincide with that of anti-malarial antibody measured by indirect fluorescent antibody test (IFAT) using parasite-infected red blood cells as antigen. Programmed death-ligand 1 (PD-L1) was up-regulated on the surface of iDCs following treatment with synthetic S100A8, not with synthetic MSP-1, AMA-1 and CSP, as compared to the expression seen for non-treated iDCs. The addition of red blood cells of infected patients to iDCs also elevated their surface expression of CD86. However, the serum levels of S100A8 decreased with increase in parasitaemia. DCs matured by sera containing S100A8 generated Treg cells from naïve T cells. The ratio of Treg cells generated was inversely proportional to the concentration of S100A8 in sera.

**Conclusions:**

Treg cells suppress the activity of cytotoxic T cells, which kill malaria parasites; therefore, the up-regulation of S100A8 in malaria patients may contribute to pathogen immune escape or tolerance.

## Background

Malaria parasites are transmitted to the host via bites from anopheline mosquitoes, and then proceed to sequentially infect hepatocytes and red blood cells (RBCs) [1]. Disease symptoms begin with blood-stage infection, and can become chronic with sustained, low levels of blood-borne parasites [2]. In the blood, malaria parasites become exposed to professional antigen-presenting cells (APCs) of the host immune system, which can then regulate the immunological response to facilitate pathogen clearance [3]. Dendritic cells (DCs) are specialized antigen-presenting cells that capture, process and present antigens to T cells [4]. The outcome of the encounter between these two cell types depends on the activation status of the DC. In the steady state, antigen presentation by DCs leads to tolerance by T cell deletion, induction of anergy, or expansion of antigen-specific CD4^+^CD25^+^Foxp3^+^ regulatory T (Treg) cells [5–8]. This sub-set of cells constitutively expresses CD25 [9], the transcription factor Foxp3 [10–12], and other T cell markers such as cytotoxic T-lymphocyte-associated protein 4 (CTLA-4) and glucocorticoid-induced TNFR-related protein (GITR) [13].

The S100 calcium binding protein A8 (S100A8), also known as myeloid-related protein 8 (MRP8) or calgranulin A, belongs to the calcium-binding S100 protein family and forms Ca^2+^-dependent heterodimeric/heterotetrameric complexes with S100A9 (MRP14/calgranulin B). Human S100A8 is 93 amino acids (aa) long (10.8 kDa) and contains two EF-hand motifs (aa 12–47 and 46–81) and one high-affinity Ca^2+^ binding site (aa 59–70). During acute and chronic inflammation, S100A8 is expressed in granulocytes, monocytes and macrophages [14] and is released from damaged cells or activated phagocytes to act as a danger signal to potentiate pro-inflammatory responses within the vascular endothelium. Thus, S100A8 is often classified as a damage-associated molecular pattern (DAMP) molecule or alarmin, which is released by activated or damaged cells under conditions of cell stress, a group that also includes S100A9, high mobility group box-1 (HMGB1) and serum amyloid A (SAA), and which are closely related to several forms of disease, including sepsis, arthritis, atherosclerosis, and cancer [15–18]. S100A8 is suggested to play a pro-inflammatory role in the innate immune response, but transitions to a regulatory function in adaptive immunity, which may be an important mechanism to avoid inflammation-induced tissue damage [19]. Furthermore, previous reports have shown that 84.7 % of patients with falciparum malaria have high levels of S100A8 in their blood [20]. *Plasmodium vivax* parasites can often obtain immune tolerance or elicit immune escape mechanisms, thereby leading to longer lifespans than *Plasmodium falciparum* parasites in human hosts in Korea. Therefore, the effects of S100A8 on infection status in patients with *P. vivax* only were investigated to better assess its role in disease progression and relapse.

## Methods

### Microscopic examination

Thick blood films were prepared to determine the infectivity of patient blood samples. The blood was fixed with methanol and stained with Giemsa diluted with buffered water at pH 7.2 to emphasize the parasite inclusions in the RBCs. To estimate the densities of blood-stage parasites, the number of asexual parasites observed in 200 white blood cells (WBCs) was totalled and the parasite : WBC ratio was multiplied by 8000 (assumed number of WBCs/μl blood) [21].

### Ethics statement

All participants, including healthy adult blood donors, were informed about the methodology and signed an informed consent form according to ethical standards set by the Human Ethics Committee of Korea National Institute of Health approved. The study was conducted according to the principles expressed in the Declaration of Helsinki, and the study procedures, potential risks and benefits were explained to all investigators. Furthermore, all the data were blinded for analysis, and all patient names were excluded.

### Detection of S100A8 in sera

The MRP8/14 (Calprotectin) Enzyme Immunoassay kit (BMA Biomedicals, Rheinstrasse, Switzerland) was used to determine the level of S100A8 in serum samples [20]. In brief, samples were diluted with assay buffer (1:100, v/v) and 100 μl was added to each well of a microtitre plate pre-coated with S100A8 monoclonal antibody. The plates were sealed and incubated for 16 h at 4 °C. Wells were washed six times with phosphate-buffered saline (PBS) prior to the immediate addition of 200 μl substrate buffer containing tetramethylbenzidine (TMB) and H_2_O_2_ solution for 2 min at room temperature. The colour reaction was stopped by adding 100 μl stop solution to each well. The absorbance was read at 450 nm with the reference wavelength set to 650 nm. Serially diluted standard solutions were run for each reaction to calculate the amount of S100A8 protein in each sample.

### Indirect fluorescent antibody test

To test for antibodies against malaria, indirect fluorescent antibody tests (IFATs) were performed with whole blood antigen against *P. vivax* [22]. Briefly, 10 ml of malaria parasite-infected blood was collected by venipuncture from *P. vivax* patients. After removing the plasma, the cells were suspended in PBS (pH 7.2) and centrifuged for 5 min at 2500 rpm. The supernatant was discarded, and the cells were resuspended in fresh PBS. The wash step was repeated three more times. Finally, an appropriate amount of PBS was added to maintain the parasite concentration at no less than 1 %. Cells were then added to each well of Teflon-coated slides and dried at room temperature for 12 h prior to storage at −70 °C. To determine the antibody titres against *P.vivax* for each patient, the antigen slides were fixed in pre-cooled acetone (−20 °C) for 10 min and washed with PBS; then, 20 µL of diluted sera (1:32–1:8192 vol/vol) in PBS was added to each well. Positive and negative controls were spotted onto each slide and incubated in a humidified chamber for 30 min at 37 °C. Reactions were stopped by washing the reacted sera with PBS. The slides then were immersed in PBS for 6 min and then dried at room temperature. Diluted FITC-conjugated anti-human IgG (Sigma, 1:32 vol/vol in PBS) was added to each well, incubated and washed using the same method described above. Several drops of buffered glycerol were added to the samples, and the slides were covered with coverslips. Slides were examined under the 40× objective of a fluorescence microscope.

### Preparation of crude RBC

To obtain the crude RBC antigen from normal and *P. vivax* infected patient’s RBC, RBCs were frozen and thawed three times in −20 °C and room temperature and then centrifuged at 12,000×*g* for 30 min at 4 °C. The resultant supernatant fluid was filtered through a 0.22 µm membrane filter (Millipore Co, Billerica, MA, USA) with syringe, and used as crude RBC antigen.

### Preparation of synthetic peptides

S100A8 peptides was synthesized by 9-fluorenylmethoxycarbonyl (Fmoc) solid phase peptide synthesis (SPPS) using ASP48S (Peptron Inc, Taejeon, Korea) and purified by the reverse phase HPLC with a Vydac Everest C18 column (250 mm × 22 mm, 10 μm). Elution was carried out with a water-acetonitrile linear gradient (2–40 %, v/v, of acetonitrile) containing 0.1 % (v/v) trifluoroacetic acid. Molecular weights of the purified peptides were confirmed using LC/MS (Agilent HP1100 series, Waldbronn, Germany). In addition, partial amino acid sequences for merozoite surface protein-1 (MSP-1; DYDVVYLKPLAGMYK) [23], apical membrane antigen 1 (AMA-1; SASDQPTQYEEEMTDYQK) [24], and circumsporozoite protein (CSP; GDRADGQPA) [25] of *P. vivax,* which are well characterized as vivax malaria vaccine candidates, were synthesized to compare the function of S100A8 in vitro assays or eliminate the background as synthesized with the same method in the same company. The S100A8 peptide (MLTELEKALNSIIDVYHKYSLIKGNFHAVYRDDLKKLLETECPQYIRKKGADVWFKELDINTDGAVNFQEFLILVIKMGVAAHKKSHEESHKE) was designed based on GenBank no. CR407674.1. SPPS involves repeated cycles of coupling-wash-deprotection-wash to remove excess reagent. In peptide synthesis, Fmoc-Glu(OtBu)-Wang resin is swollen by dichloromethane (DCM) for 30 min at room temperature, after which the DCM is extracted and the resin is washed with dimethylformamide (DMF) several times. The amino fmoc group is then deprotected by piperidine, washed by DMF, and reacted with the carbonyl group of the next amino-protected amino acid Fmoc-Val-OH, with the help of coupling reagents and activator for 30 min. Kaiser tests are used to detect free amino groups. When the solution did not change colour, the previous procedure was repeated. This cycle is repeated to form the desired peptide chain. After all reactions are complete, the synthesized peptide is cleaved from the resin with reagent K, precipitated with the addition of cold diethyl ether (DEE), washed with cold DEE, and dried in vacuum to a constant weight. Electrospray ionization mass spectroscopy (ESI–MS) was performed in a WATERS ZQ2000 instrument to detect the crude sample prior to purification by HPLC (Beijing Chuang Xin Tong Heng Science and Technology Co. Ltd, LC3000).

### DC generation and maturation test

Buffy coats were obtained from healthy donors according to the institutional guidelines of Korea National Institute of Health. Peripheral blood mononuclear cells (PBMCs) were prepared by density centrifugation using Ficoll-Paque gradients (Amersham Pharmacia Biotech, Uppsala, Sweden). The PBMCs were mixed with micro-bead-conjugated anti-CD14 antibody (Miltenyi Biotech, Auburn, CA, USA) and then incubated on ice for 30 min. After washing with binding buffer (2 % FBS, 2 mM EDTA, 20 mM PBS, pH 7.4), CD14^+^ cells were purified by autoMACS (Miltenyi Biotech). The purified cells were subsequently cultured in six-well plates in RPMI 1640 medium supplemented with 10 % heat-inactivated FBS, 10 mM HEPES, 2 mM l-glutamine, 100 U/ml penicillin, 10 µg/ml streptomycin, 50 μM 2-mecaptoethanol, 5 ng/ml granulocyte–macrophage colony-stimulating factor (GM-CSF; R&D Systems, Minneapolis, MN, USA), and 10 ng/ml IL-4 (R&D). To test the effect of S100A8 on DC maturation, immature DCs (iDCs) were harvested on day 3 and cultured with synthetic S100A8, CSP, MSP-1, AMA-1, and lipopolysaccharide (LPS) for 48 h. DC maturation was assessed by the presence of CD86, PD-L1 and HLA-DR on the DC surface, as determined by fluorescence-activated cell sorting (FACS) analysis using a FACSCalibur instrument (BD Biosciences, San Jose, CA, USA).

### Treg cell induction by mature DCs

To isolate naïve T cells (CD4^+^CD25^−^Foxp3^−^), PBMCs were prepared from 30 ml of peripheral blood from healthy volunteers using density centrifugation by Ficoll-Paque gradients. Naïve T cells were isolated using a human naïve CD4^+^ T cell isolation Kit II (Miltenyi Biotech) according to the manufacturer’s instructions and separated on an AutoMACS sorter (Miltenyi Biotech). The unlabelled CD4^+^CD25^−^ population (>98 % pure) was then used for Treg cell generation assays. To generate Treg cells, synthetic peptide-matured DCs and blood samples were cultured in 96-well plates at 1 × 10^6^ cells/ml in culture medium (RPMI 1640 supplemented with 20 % heat-inactivated FBS, 2 mM l-glutamine, 100 U/ml penicillin, 10 µg/ml streptomycin) and stimulated with anti-CD3 (OKT3, 200 ng/ml; Janssen-Cilag, Raritan, NJ, USA) and soluble anti-CD28 (200 ng/ml; BD Biosciences) monoclonal antibodies in the presence of 100 U/ml rIL-2 and analysed 5 days later. Cells activated similarly in the presence of solely rIL-2 or in the absence of any synthetic peptides and blood samples served as controls. In some experiments, cells were stained with the fluorescent dye carboxyfluorescein succinimidyl ester (CFSE) (Life Technologies, Grand Island, NY, USA), prepared using the manufacturer’s protocol, to monitor proliferation.

### Flow cytometric analysis

iDCs were stimulated with LPS, synthetic S100A8, MSP-1, CSP and AMA-1 peptides, blood, and sera of malaria patients or healthy controls at 37 °C for 48 h. Induced mature DCs were then incubated with MHC class-II (HLA-DR)-FITC, CD86-PE, and PD-L1-APC specific antibodies on ice for 15 min, washed twice in staining buffer (PBS (pH 7.2) supplemented with 2 % FBS and 0.1 % sodium azide), and fixed in PBS containing 1 % paraformaldehyde. CD4^+^ T cells were incubated with CD3-Alexa Fluor 700, CD4-PE, and CD25-PE/Cy5 specific antibodies for 20 min at room temperature and washed with staining buffer. Intracellular staining for Foxp3 was performed following the manufacturer’s recommendations (BD Pharmingen). Briefly, the cells were incubated with 500 µl of 1× permeabilizing solution 2 (BD Biosciences) for 10 min at room temperature in the dark, washed with staining buffer, and then incubated with Foxp3-APC antibody for 20 min at room temperature. After two additional washes in staining buffer, cells were fixed in PBS containing 1 % paraformaldehyde. Isotype-matched control antibodies were included for all experiments as controls for nonspecific binding. The dead cells were gated out by forward and side scatter. Flow cytometry was performed using a FACSCalibur (BD Bioscience) instrument, and the data were analysed using FlowJo software (TreeStar, Ashland, OR, USA).

### Measurement of cytokine levels

Plasma was obtained from vivax malaria patients and centrifuged for 15 min at 2500 rpm; 200 μl aliquots were stored at −80 °C until use. Cytokine concentrations were measured in duplicate using a Bio-Plex Pro human cytokine multiplex assay kit (Bio-Rad, Hercules, CA, USA) according to the manufacturer’s instructions.

### Statistical analysis

Data analysis was performed using GraphPad Prism version 4.0 (GraphPad Software, San Diego, CA, USA). All values have been expressed as the mean ± standard deviation (SD). A *P* value of <0.05 was considered statistically significant. The relationship between sera S100A8 concentrations and reciprocal antibody titer was compared using one-way ANOVA followed by the Kruskal–Wallis test. The induction of mature DCs by LPS and synthetic peptides and the cytokine production of these cells were compared using one-way ANOVA followed by Bonferroni’s multiple comparison tests. DC maturation by patient sera and crude patient RBC antigens were compared using two-way ANOVA. The effect of synthetic peptides on Treg cell induction and S100A8 levels in sera among malaria patients and healthy controls were compared by t-test followed by Mann–Whitney test. The correlations between S100A8 serum concentration and parasitaemia in malaria patients on Treg cell induction were analysed by linear regression.

## Results

### Level of S100A8 in sera from *Plasmodium vivax*- infected patients

**Fig. 1:**
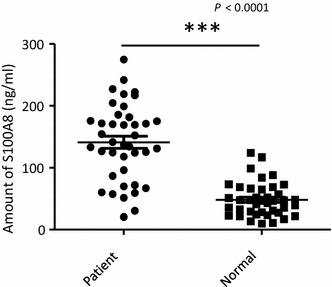
Expression of S100A8 in *Plasmodium vivax*-infected patients. Comparison of S100A8 concentrations in the sera of patients and healthy controls. The concentrations of S100A8 in blood samples were measured with a MRP8/14 Enzyme Immunoassay kit

**Fig. 2:**
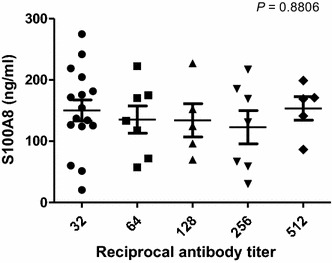
Expression of S100A8 in *Plasmodium vivax*-infected patients. Relationship between S100A8 concentration and reciprocal antibody titer in sera against *P. vivax*. Antibody titres were measured by the immunofluorescence antibody test (IFAT)

**Fig. 3:**
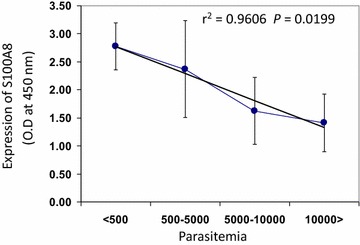
Expression of S100A8 in *Plasmodium vivax*-infected patients. S100A8 expression in patients with *P. vivax*, according to their parasitaemia. A *P* value of <0.05 was considered statistically significant. The relationship between S100A8 concentrations and reciprocal antibody titre in sera was compared using one-way ANOVA followed by the Kruskal–Wallis test. S100A8 expression in patients according to parasitaemia was analysed by linear regression

It is not known whether vivax malaria patients exhibit increased serum concentrations of S100A8, as seen with falciparum malaria patients [20]. Therefore, the concentrations of S100A8 in 40 malaria patients who were confirmed to be infected with Korean isolates of *P. vivax* was first measured and compared with those of 40 healthy controls (Fig. 1). The mean level of S100A8 detected was 141.2 ± 61.849 ng/ml in *P. vivax*-infected patients and 48.1 ± 27.384 ng/ml in the normal control group (*P* < 0.0001). To determine if any correlation existed between S100A8 concentrations and anti-malaria antibody titres, IFAT assays using *P. vivax*-infected RBC antigen were used to assess the presence of anti-malaria antibodies in the sera. Interestingly, the level of S100A8 did not coincide with that of anti-malaria antibodies (Fig. 2, *P* = 0.8806). It was expected that S100A8 concentration might correlate with parasitaemia; however, S100A8 concentrations were significantly decreased in patients with increased levels of parasitaemia (Fig. 3, *P* = 0.0199).

### Function of S100A8 in DC maturation

**Fig. 4:**
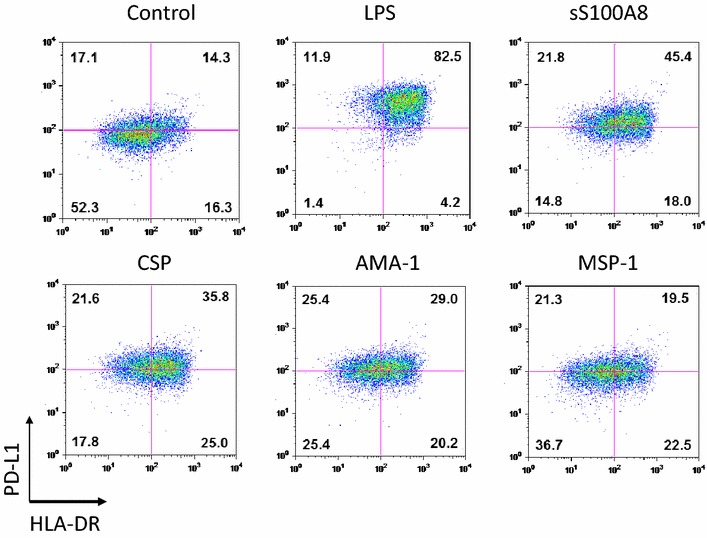
Comparison of the DC maturation state between LPS and synthetic peptides, S100A8, CSP, AMA-1, and MSP-1. The maturation state of immature DCs was assessed by the expression levels of PD-L1 and HLA-DR on the DC surface, as determined by FACS analysis

**Fig. 5:**
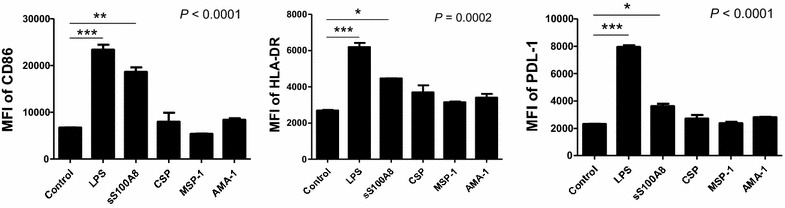
Comparison of the DC maturation state between LPS and synthetic peptides, S100A8, CSP, AMA-1, and MSP-1. DC maturation as determined by the expression levels of CD86, HLA-DR, and PDL-1 by several synthetic peptides. The relative increase in surface expression is expressed as the mean fluorescence intensity (MFI) of matured DCs over the MFI of iDCs. A representative result of three repeated experiments is shown for FACS analysis. Significant differences were determined using one-way ANOVA followed by Bonferroni’s multiple comparison tests (*P < 0.05, **P < 0.01, ***P < 0.001)

**Fig. 6:**
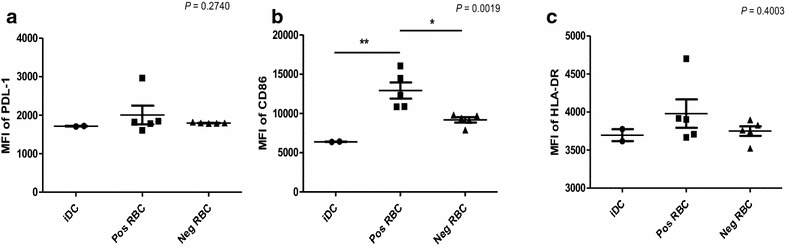
DC maturation by crude antigens of red blood cell infected with *Plasmodium vivax. iDC* untreated control DC, *Pos RBC* malaria positive red blood cell treated DC, *Neg RBC* normal healthy volunteer’s red blood cell treated DC. DC maturation was assessed by the levels of **a** PD-L1, **b** CD86 and **c** HLA-DR on the DC surface as measured by fluorescence-activated cell sorting (FACS). The relative increase in surface expression is expressed as the mean fluorescence intensity (MFI) of matured DCs over the MFI of iDCs. A representative result of three repeated experiments is shown. Significant differences were determined using the two-way ANOVA (*P < 0.05, **P < 0.01, n = 5)

To determine the effect of S100A8 on DC maturation, immature myeloid DCs were generated by culturing CD14^+^ PBMCs in the presence of GM-CSF and IL-4 for 3 days, followed by incubation with synthetic S100A8, MSP-1, AMA-1, CSP, and LPS for 48 h. Expression of the DCs’ maturation markers, CD86, HLA-DR and PD-L1, were then assessed by flow cytometric analysis. When comparing the expression levels of HLA-DR and PD-L1, 14.3 % of the untreated controls were found to be double-positive, whereas LPS induced high levels of expression on the iDC surface, with 82.5 % of cells being double-positive, followed by 45.5 % cells showing positivity for S100A8, 35.8 % for CSP, 29.0 % for AMA-1, and 19.5 % for MSP-1 (Fig. 4). LPS (*P* < 0.001) and synthetic S100A8 (*P* < 0.05) displayed significant elevations of CD86, HLA-DR and PD-L1; however, the CSP, MSP-1 and AMA-1 synthetic peptides were found to have no significant effect on induction on comparison with control, untreated iDCs (Fig. 5). The effect of parasite-infected RBCs (Fig. 6) containing S100A8, as verified by ELISA, were then tested on DC maturation. Notably, infected RBCs’ crude antigen significantly increased the expression of CD86 on iDCs (*P* = 0.0019, n = 5, Fig. 6b). It means that some components of malaria parasites may directly affect DC maturation or induce the expression of S100A8 in iDC, which is used in DC maturation as self-feeding manner to be matured DC.

### Mature DCs generate Treg cells

**Fig. 7:**
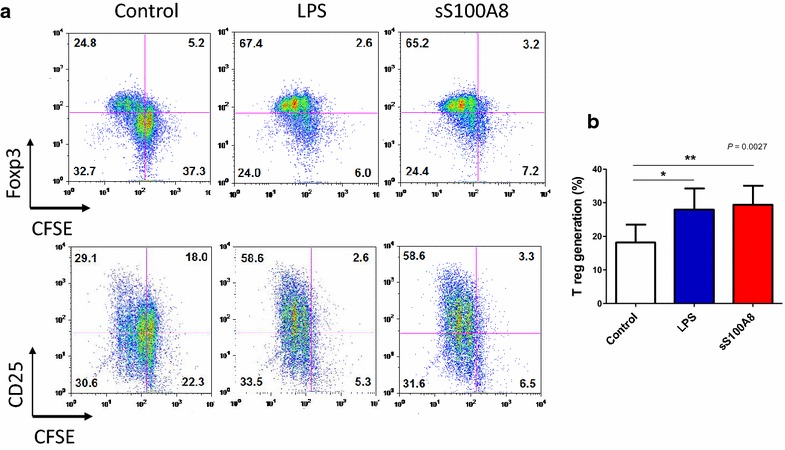
CD4^+^CD25^+^Foxp3^+^ regulatory T (Treg) cell generation by synthetic S100A8. Naïve T cells (CD4^+^CD25^−^Foxp3^−^) were isolated from the peripheral blood of healthy volunteers and stimulated with anti-CD3 and anti-CD28 monoclonal antibodies in the presence of 100 U/ml recombinant interleukin-2 (rIL-2) and/or synthetic peptides for 5 days. To monitor the cell proliferation, naïve T cells were stained with the fluorescent dye carboxyfluorescein succinimidyl ester (CFSE). Treg cell generation was evaluated by FACS analysis. **a** A representative result of FACS profile of Treg generation by LPS and sS100A8. **b** Significantly increased Treg generation by LPS and sS100A8 obtained by seven separate experiments with different volunteers’ DC each. Significant differences were determined using the one-way ANOVA (*P < 0.05, **P < 0.01, n = 5)

**Fig. 8:**
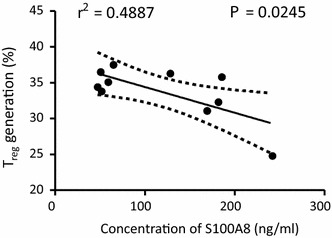
Treg cell induction in the presence of S100A8-containing sera. DCs matured in the presence of sera of malaria patients were co-cultured with naïve T cells stained with CFSE. Treg cell generation was evaluated by FACS analysis and analysed by linear regression

**Fig. 9:**
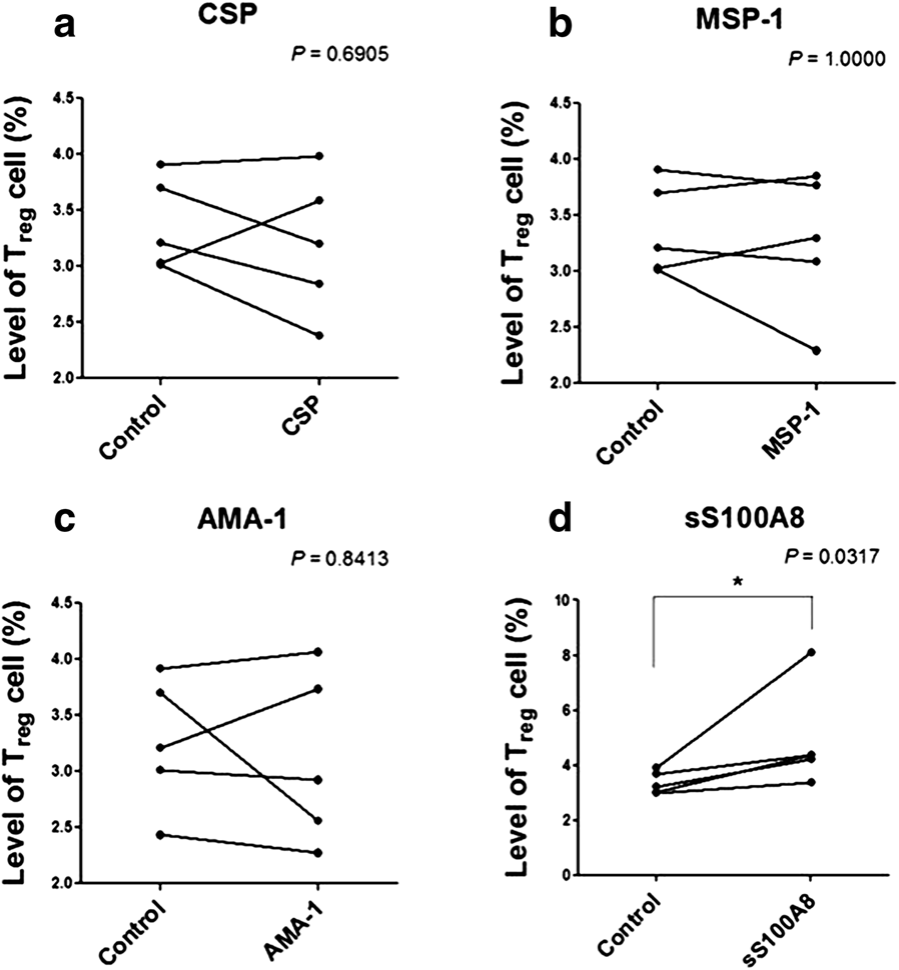
Increased Treg cell induction in normal PBMCs on adding synthetic **a** CSP, **b** MSP-1, **c** AMA-1, **d** S100A8. PBMCs from healthy volunteers were cultured in the presence of 1 μg of each synthetic peptide and cultured for 5 days. Treg cell induction was then evaluated by FACS analysis. Significant differences were determined using the Mann–Whitney test (*P < 0.05, n = 5)

The combined blockade of PD-L1 and lymphocyte-activation gene 3 (LAG-3) accelerates the clearance of yoelii malaria and correlates with improved CD4^+^ T cell and B cell responses [26]. However, since PD-L1 can interact specifically with both B7-1 [27] and PD-1 [28] to inhibit T cell activation, either pathway—individually or cooperatively—could contribute to the attrition of malarial immunity. Therefore, further analysis of the functional status of matured DCs in healthy volunteers was examined by measuring their capability to induce Treg cells. Significantly, the presence of LPS (67.4 %) and S100A8 (65.2 %) induced Treg cells as much as two times higher than non-treated control cells (24.8 %, Fig. 7a). When Treg generation was tested with seven volunteers’ DC, control group showed Treg generation as 18 ± 14.13 % and sS100A8-treated group (29.40 ± 14.90 %) showed higher than LPS-treated group (27.93 ± 16.70 %) (Fig. 7b). Furthermore, naïve T cells cultured with S100A8-containing sera led to Treg cell generation that was inversely proportional to the concentration of S100A8 in the sera (Fig. 8, *P* = 0.0245, n = 10). Interestingly, synthetic S100A8 always increased the induction of Treg cells in PBMCs isolated from healthy volunteers (Fig. 9d, *P* = 0.0465), but was not constant in synthetic MSP-1, AMA-1 and CSP treatments (Fig. 9a–c).

### Cytokine production by mature DCs

**Fig. 10:**
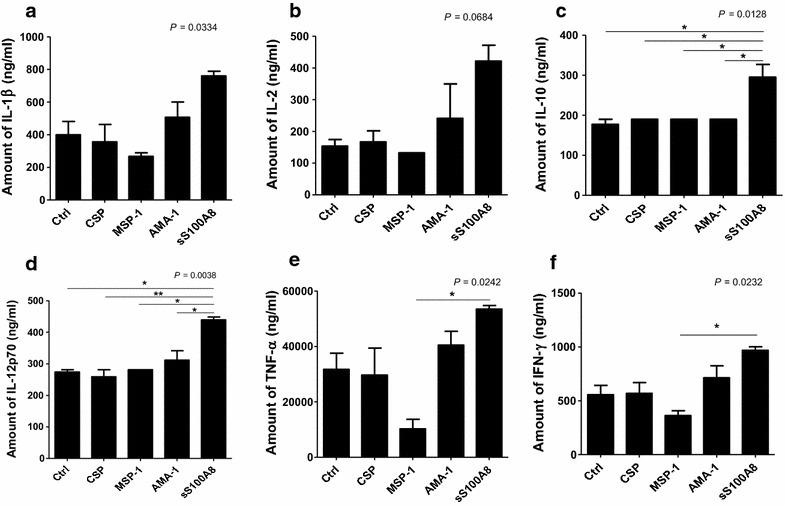
Comparison of cytokine production in DCs matured in the presence of synthetic peptides. iDCs were stimulated with synthetic peptides for 48 h and each supernatant was applied to Bio-Plex Pro human cytokine multiplex assay plates. Ctrl; not treated iDC, CSP; synthetic circumsporozoite protein treated iDC, MSP-1; synthetic merozoite surface protein-1 treated iDC, AMA-1; synthetic apical membrane antigen-1 treated iDC, sS100A8; synthetic S100A8 treated iDC. Expression level of **a** IL-1β, **b** IL-2, **c** IL-10, **d** IL-12p70, **e** TNF-α, and **f** IFN-γ in matured DC by several synthetic peptides. Data were compared using one-way ANOVA followed by Bonferroni’s multiple comparison tests. The experiment was performed in duplicate (*P < 0.05, **P < 0.01)

IL-10 and IL-12p70 were significantly elevated in mature DCs following treatment with synthetic S100A8, compared to other vaccine candidate peptides (Fig. 10c, d, *P* = 0.0128 and *P* = 0.0038, respectively). IL-1β, IL-2, tumour necrosis factor (TNF), and interferon-γ (IFN-γ) also increased, but the differences were not statistically significant (Fig. 10a, b, e, f).

## Discussion

The severity of malaria can range from asymptomatic to lethal infections involving severe anaemia and cerebral disease; however, the molecular and cellular factors responsible for these differences are poorly understood. Thus, identifying the factors that mediate virulence will contribute to developing antiparasitic therapy. Similar to the findings for other infectious diseases, DCs are essential for initiating adaptive and innate immune responses in malaria. Although DCs are known to be important for initiating immunity to malaria [29, 30], studies describing the function of DCs in the adaptive response to infection have not explored their direct role in disease outcome, survival, anaemia, or parasitaemia. Furthermore, limited data exist on the differing outcomes of *Plasmodium* infections. Interestingly, the mean incubation period of Korean *P. vivax* isolates is reported to extend as long as 279 ± 41 days (range 153–452 days) [31]. The ratio of cases with short and long incubation periods was 25 and 75 %, respectively [32]. As such, it was hypothesized that disease in patients with long incubation periods might be due to pathogens that have undergone immune escape or tolerance. It is mainly attributable to Treg cells that have a crucial role in immune suppression during malaria infection, as well as the escape of parasites from host immune surveillance [33, 34].

Two main DC sub-sets have been characterized in humans, myeloid (mDC, CD11c+) and plasmacytoid DCs (pDCs, CD11c^−^) [35]. A sub-set of mDCs (also known as monocyte-derived DCs, MDDCs or mo-DCs) are differentiated from monocytes (CD14^+^) when cultured in the presence of GM-CSF and IL-4 [36]. DCs express a wide repertoire of membrane receptors, including pattern recognition receptors (PRRs) that induce their terminal differentiation and activation upon engagement by DAMPs or pathogen-associated molecular patterns (PAMPs) [37]. C-type lectin receptors (CLRs), a sub-type of PRRs, promote the activation of Syk and CARD9 and preferentially induce the polarization of naïve Th0 lymphocytes into Th17 cells [38–40]. In comparison, tolerogenic DCs are able to inhibit the differentiation of naïve Th0 lymphocytes, thus suppressing the generation of T cell-mediated immune responses, and induce the generation of Treg cells [41, 42]. In this regard, it has been theorized that different membrane receptors (e.g., PSGL-1 and PD-L1), as well as cytokines (e.g., IL-10), are able to induce the generation of tolerogenic DCs [43, 44]. Since S100A8 is reported to promote the generation of Treg cells through the induction of tolerogenic DCs [45], the role of S100A8 was investigated in *P. vivax* patients. Notably, the amount of S100A8 in vivax malaria patients was three times higher than that in healthy volunteers (Fig. 1), and the mean value of S100A8 produced by vivax malaria patients (141.2 ng/ml) was less than that of falciparum malaria patients (3420 and 13,580 ng/ml for parasitaemia <0.2 and ≥0.2 %, respectively) [20]. However, these levels did not correlate with the amount of antibodies produced (Fig. 2). Interestingly, antibody titres were inversely correlated with parasitaemia (Fig. 3); however, the level of serum S100A8 was significantly related to the parasitic load in patients with *P. falciparum*, as well as fever episodes in children up to 6 years of age [20]. This observation emphasizes a potential difference in the function of S100A8 in *P. vivax* and *P. falciparum* patients. Comparison of the induction of PD-L1 and HLA-DR double-positive iDCs by several vaccine candidate peptides and synthetic S100A8 showed that approximately half of the double-positive cells were induced by synthetic S100A8 compared to LPS. The Pv210 type of synthetic CSP induced double-positive DCs with relatively higher expression than that of AMA-1 and MSP-1 (Fig. 4). This suggests that CSP might induce immune tolerance in some patients, but not in a statistically significant manner (Fig. 5). As expected, parasite-infected RBCs from vivax malaria patients significantly induced DC maturation than in normal persons (Fig. 6). However, *P. falciparum*-infected RBC appeared to inhibit DC maturation, subsequently reducing its capacity to stimulate T cells [46]. Therefore, S100A8 in vivax malaria appears to promote its immune escape, as elevations in PD-L1 on the iDCs surface by synthetic S100A8 promotes Treg cell induction greater than that observed in controls (Fig. 7). The serum levels of S100A8 decreased with increase in parasitaemia (Fig. 3) and the ratio of Treg cells generated was inversely proportional to the concentration of S100A8 in sera (Fig. 8). This means that S100A8 function is controlled by unknown factors in the sera for maintaining the immune balance or S100A8 concentration may reflect the beginning of onset. Therefore, the next goal is to investigate these factors or investigation of S100A8 concentration according to time course of disease onset which affect the Treg generation rate. Only synthetic S100A8 was sufficient to increase Treg cell induction from healthy volunteer PBMCs, whereas the CSP, MSP-1 and AMA-1 synthetic peptides were unable to mediate this effect (Fig. 9). In addition, IL-10 production in iDCs increased significantly with the addition of synthetic S100A8 (Fig. 10). The immunoregulatory mechanisms involving IL-10 appear complex and involve both pro-inflammatory and suppressive functions, although the prime function of IL-10 is to protect the host from excessive immune and inflammatory responses [47]. Nevertheless, S100A8 plays a clear pro-inflammatory function by facilitating the secretion of IL-1β, IL-12p70, TNF-α, and IFN-γ by mature DCs (Fig. 10). In the mouse, S100A8 acts as a potent chemo-attractant for neutrophils and monocytes in vitro and in vivo [48–50] to influence leukocyte migration and transmigration into tissues by increasing their deformability [51]. Therefore, S100A8 likely plays dual roles in the inflammatory response depending upon the environmental conditions. Thus, synthetic S100A8 up-regulated the expression of HLA-DR, PD-L1, CD86, and IL-10 in DCs necessary for generating Treg cells, which contributes to escape from the host immune defences [52]. The presence of parasite-specific CD4^+^ T cells correlates with reduced parasite burdens and disease severity in malaria, but the effect of parasites on Treg cell induction is the opposite [28]. The source of S100A8 in malaria remains unclear, although it is known to be secreted from monocytes, macrophages and neutrophils [53–58]. Petersen et al. found that a novel mechanism of immune regulation mediated by S100A8 is an important factor in orchestrating coordinated immune reactions. This observation allows for the propagation of an appropriate innate inflammatory response by acting as a classical DAMP protein, while also preventing hyper-activation of the adaptive immune system so as to avoid the tissue damage that can be caused by excessive immune responses [19]. S100A8 may exhibit pleiotropic effects and promote IL-10 secretion to elicit synergic effects that regulate harmful inflammatory tissue damage by virtue of its capacity to act as an antioxidant [59], thereby protecting against acute cytokine-mediated pathology.

## Conclusions

These findings suggest that S100A8 provides an immune environment friendly toward vivax malaria parasites by elevating Treg, which suppress the parasite-activated T cells. Therefore, this kind of environmental change may contribute to the long-term persistence of *P. vivax* in human host.
