# Erratum to: Up-regulated S100 calcium binding protein A8 in *Plasmodium*-infected patients correlates with CD4^+^CD25^+^Foxp3 regulatory T cell generation

**DOI:** 10.1186/s12936-015-1034-3

**Published:** 2015-12-17

**Authors:** Tong-Soo Kim, Yoon-Joong Kang, Jung-Yeon Kim, Sangeun Lee, Won-Ja Lee, Youngjoo Sohn, Hyeong-Woo Lee

**Affiliations:** Department of Parasitology and Tropical Medicine, Inha Research Institute for Medical Sciences, Inha University School of Medicine, Incheon, 400-712 Republic of Korea; Department of Biomedical Science, Jungwon University, Goesan, Chungbuk 367-805 Republic of Korea; Division of Malaria and Parasitic Disease, Korea National Institute of Health, Osong, 363-951 Republic of Korea; Department of Pathology, Immunology, and Laboratory Medicine, College of Medicine, University of Florida, Gainesville, FL 32610 USA; Division of Arbovirus, Korea National Institute of Health, Osong, 363-951 Republic of Korea; Department of Anatomy, College of Korean Medicine, Institute of Korean Medicine, Kyung Hee University, Seoul, 130-701 Republic of Korea

## Erratum to: Malar J (2015) 14:385 DOI 10.1186/s12936-015-0855-4

After publication of the original article [1], it was noticed that the order of the author list was incorrect. Due to the revision of the submission prior to editorial acceptance, Production received a version of the manuscript with an incorrect order given, with Hyeong-Woo Lee mistakenly listed as first author.

Both Tong-Soo Kim and Yoon-Joong Kang contributed equally as first authors of the article, so the author list should have appeared as follows:

Tong-Soo Kim^1†^, Yoon-Joong Kang^1†^, Jung-Yeon Kim^3^, Sangeun Lee^4^, Won-Ja Lee^5^, Youngjoo Sohn^6*^ and Hyeong-Woo Lee^4*^

^†^Tong-Soo Kim and Yoon-Joong Kang contributed equally to this work as first author.
